# An evaluation tool for backbone extraction techniques in weighted complex networks

**DOI:** 10.1038/s41598-023-42076-3

**Published:** 2023-10-09

**Authors:** Ali Yassin, Abbas Haidar, Hocine Cherifi, Hamida Seba, Olivier Togni

**Affiliations:** 1 Laboratoire d’Informatique de Bourgogne, University of Burgundy, Dijon, France; 2 Computer Science Department, Lebanese University, Beirut, Lebanon; 3 ICB UMR 6303 CNRS, Univ. Bourgogne - Franche-Comté, Dijon, France; 4 UCBL, CNRS, INSA Lyon, LIRIS, UMR5205, Univ Lyon, 69622 Villeurbanne, France

**Keywords:** Complex networks, Statistical physics, Computer science, Software, Computational science

## Abstract

Networks are essential for analyzing complex systems. However, their growing size necessitates backbone extraction techniques aimed at reducing their size while retaining critical features. In practice, selecting, implementing, and evaluating the most suitable backbone extraction method may be challenging. This paper introduces netbone, a Python package designed for assessing the performance of backbone extraction techniques in weighted networks. Its comparison framework is the standout feature of netbone. Indeed, the tool incorporates state-of-the-art backbone extraction techniques. Furthermore, it provides a comprehensive suite of evaluation metrics allowing users to evaluate different backbones techniques. We illustrate the flexibility and effectiveness of netbone through the US air transportation network analysis. We compare the performance of different backbone extraction techniques using the evaluation metrics. We also show how users can integrate a new backbone extraction method into the comparison framework. netbone is publicly available as an open-source tool, ensuring its accessibility to researchers and practitioners. Promoting standardized evaluation practices contributes to the advancement of backbone extraction techniques and fosters reproducibility and comparability in research efforts. We anticipate that netbone will serve as a valuable resource for researchers and practitioners enabling them to make informed decisions when selecting backbone extraction techniques to gain insights into the structural and functional properties of complex systems.

## Introduction

In recent years, the exponential growth of available data has prompted a surge in studying complex systems across various research domains. Networks have become a standard tool for modeling the entities and their interactions within such systems, with nodes and edges representing the entities and their relationships, respectively^1–10^. Moreover, the toolbox for network analysis continues to expand, with the introduction of numerous tools to facilitate various network analysis tasks^11–34^. However, analyzing large networks can be challenging. One solution to this issue is reducing the network size while retaining its essential properties. This objective is an active research area referred with various terms in the literature, such as sparsification, summarization, validated network extraction, skeleton extraction, and backbone extraction^35–67^.

Backbone extraction offers several advantages, including reduced data volume and storage, faster graph algorithms and queries, support for interactive analysis, and noise elimination. Backbone extraction has a wide range of applications. One uses it for various types of networks, such as social^68–76^, biological^77^, brain^78–81^, gene^82–86^, metabolic^87–91^, food web^92,93^, environmental^94,95^, finance^96–99^, trade^100–104^, information^105–108^, political^109,110^, transportation^111–115^ , and others^116–119^. These applications have a broad range of uses, including clustering, classification, community detection, outlier detection, pattern set mining, identification of sources of infection in large graphs, and visualization, among others.

Practitioners must operate the most suitable method for their various applications or use cases. Therefore, there has been a growing interest in comparing backbone extraction techniques in the literature^120–125^. Furthermore, new tools have been introduced to fulfill the multitude of applications requirements^29,35^. These tools implement a variety of backbone extraction techniques in different frameworks.

One can distinguish two main backbone extraction approaches: structural and statistical. Structural methods focus on the network’s topological features to extract a backbone with specific structural properties. They remove nodes or edges less critical for the properties to preserve. In contrast, statistical methods aim at eliminating noisy nodes or edges that blur the network information. They evaluate the significance of nodes and edges using a hypothesis-testing framework. They remove nodes or links qualified as noise.

The tool introduced by Coscia in^35^ incorporates three statistical and three structural backbone-extracting methods for weighted networks. Its Python module uses pandas^126^ to enhance the performance. The backbone extraction techniques operate on a DataFrame input. They can process directed and undirected networks.

Neal presents backbone an R package to extract network backbones in^29^. It implements seventeen backbone extraction methods. Six methods are primarily designed for bipartite projections, two for weighted networks, and ten for unweighted networks. It also provides the generic sparsify() function that allows the custom construction of many more backbone methods. The methods operate on a R Matrix object, sparse Matrix object, a DataFrame object, or an igraph as input. They allow the processing of directed and undirected networks.

Traditionally, users need to implement their code to compare different backbone extraction methods. We introduce netbone, a Python package specifically designed for extracting and comparing backbones from simple weighted networks. It offers an extensive collection of methods, including six statistical, thirteen structural, and one hybrid backbone extraction methods. It also provides filtering flexibility to tailor the backbone extraction process. Furthermore, it implements multiple ways to compare backbones.

They include a boolean filter for extraction techniques that extract a single backbone. Threshold and fraction filters are dedicated to methods assigning scores to the nodes or links. Users can indicate a threshold value to filter out elements with scores below its value. They can also show the fraction of features preserved associated with the top scores. In addition, the package provides a comparison framework with a visualization module to plot the results. Its goal is to assist the users in comparing various backbone extraction methods using a set of evaluation measures. This framework allows comparing backbone properties, distributions, and the evolution of various network properties when the backbone size is tunable. The package includes a set of predefined properties used for evaluating the extracted backbones. Moreover, users can integrate their backbone extraction methods and evaluation measures into the comparison framework. Furthermore, the framework facilitates the extraction of the consensual backbone, characterized by the common nodes and edges among a given set of backbones.

In the following sections, first, we briefly introduce the backbone extraction methods implemented in the netbone package. Then we present the package architecture and its modules, highlighting its numerous advantages. Next, we provide a simple toy example illustrating how to use netbone. Finally, we showcase the power of netbone’s comparison framework through five experiments. The first experiment illustrates how the comparison framework can assist in evaluating the backbone extraction methods by comparing various topological properties. The second experiment highlights how the framework could aid users in determining the appropriate fraction or threshold for extracting backbones. The third experiment illustrates how users evaluate the distribution of property values of the extracted backbones. The fourth experiment introduces the consensual backbone and how users can create unlimited combinations using the backbone extraction methods. Finally, the fifth experiment illustrates how users can integrate their new backbone extraction method into the comparison framework and evaluate it using their custom evaluation measures.

## Backbone extraction methods

Backbone extraction methods identify the most significant or essential parts within a network. Edge filtering techniques capture the most important relationships between nodes while removing less meaningful or noise-like connections. However, defining a crucial link in a network can be subjective and dependent on the specific application or research question. Therefore, researchers have developed several approaches to identify and extract the backbone of a network, each with its assumptions and criteria for importance. One can distinguish mainly statistical and structural methods. Additionally, hybrid methods incorporate both statistical and structural approaches. Table 1 summarizes the main features of methods implemented in netbone.

### Statistical backbone extraction methods

Statistical backbone methods evaluate the significance of edges in a network using hypothesis testing based on empirical distribution or a null model. They compute p-values for each edge and filter edges based on their p-values. netbone implements six statistical backbone filtering techniques:

- **Disparity Filter**^45^: It assumes that the normalized weights of a node’s edges follow a uniform distribution. Comparisons of the observed normalized edge weights to this null model allow filtering out edges at a desired significance level $\alpha$. Since we define a null model for each node, an edge weight can be significant from the viewpoint of one of its nodes and not the other.
- **Marginal Likelihood Filter**^52^: While the Disparity Filter assess the significance of an edge in the light of each node it connects independently, the Marginal Likelihood filter considers the two nodes the edge connects. It assumes an integer-weighted link as multiple unit edges. The null model assumes that each unit edge randomly chooses two nodes, which results in a binomial distribution. In other words, it calculates the probability of drawing at least *w* unit edges from the strength of the network (summation of all weights) with probability proportional to both nodes’ strengths.
- **Noise Corrected Filter**^35^: Like the Marginal Likelihood Filter, it assumes edge weights are drawn from a binomial distribution. However, using a Bayesian framework, it estimates the probability of observing a weight connecting two nodes. This framework enables us to generate posterior variances for all edges. This posterior variance allows us to create a confidence interval for each edge weight. Finally, we remove an edge if its weight is less than $\delta$ standard deviations stronger than the expectation ($\delta$ is the only parameter of the algorithm). It also provides a direct approximation through Binomial distribution similar to the Marginal Likelihood Filter.
- **Enhanced Configuration Model Filter**^51^: It Enhances the null model of the Marginal Likelihood filter. Using the Enhanced Configuration Model of network reconstruction, its null model is based on the canonical maximum-entropy ensemble of weighted networks with the same degree and strength distribution as the actual network.
- **Locally Adaptive Network Sparsification Filter**^55^: It makes no assumptions about the underlying weight distribution. Instead, the empirical cumulative density function is used to evaluate the statistical significance. Thus, from the viewpoint of edge incident nodes, it calculates the probability of choosing an edge randomly with a weight equal to the observed weight.
- **Multiple Linkage Analysis**^60^: It assumes that the weights are evenly distributed among the node’s neighbors, ranging from 1 to n. It calculates a goodness of fit by comparing the observed distribution with the hypothetical ones using a correlation coefficient. The optimal number of edges to retain for each node is determined by the number that yields the highest correlation coefficient.

### Structural backbone extraction methods

Structural backbone methods operate on the network’s topology to extract a backbone with specific topological properties. One can divide them into two categories. The first category includes techniques for extracting a single substructure from the network. They cannot be adjusted and typically result in a single backbone. The second category assigns scores to nodes or edges based on topological features. These methods can be tuned by setting a threshold $\beta$ or selecting the top fraction of scores. Netbone contains thirteen structural backbone filtering techniques:

- **Global Threshold Filter:** It is the most straightforward technique. It filters edges with weights lower than a predefined threshold $\beta$.
- **Maximum Spanning Tree Filter:** It extracts a subgraph that includes all the nodes connected without forming cycles with the maximum total edge weight.
- **Doubly Stochastic Filter**^54^: It transforms the network’s adjacency matrix into a doubly stochastic matrix by iteratively normalizing the row and column values using their respective sums. Next, one sorts the edges in descending order based on their normalized weight. One adds the edges to the backbone sequentially until it includes all nodes in the original network as a single connected component. It is not always possible to transform the matrix into a doubly-stochastic one.
- **High Salience Skeleton Filter**^56^: It is based on the concept of edge salience. First, one constructs a shortest path tree for each node by merging all the shortest paths from that node to every other node in the network. Then the edge salience is computed as the proportion of shortest-path trees where the edge is present. The authors observed that edge salience follows a bimodal distribution near the boundaries 0 and 1. Consequently, they retain only the edges with salience near 1, eliminating the need to select an arbitrary threshold.
- **h-Backbone Filter**^41^: It is inspired by the h-index and edge betweenness. First, using the edge weights, it extracts the h-strength network: h is the largest natural number such that there are h links, each with a weight at least equal to h. Then it extracts the h-bridge network similarly. A bridge of an edge is the edge betweenness divided by the number of all nodes. Finally, the h-backbone merges the two networks.
- **Metric and Ultrametric distance backbone filters**^44^: Both methods extract a subgraph consisting of the shortest paths in the network. Still, they diverge in their definitions of the shortest path length. Specifically, the Metric filter defines the shortest path length as the sum of the edge distances. In contrast, the Ultrametric filter defines it as the maximum distance among all edges in the path.
- **Modularity Backbone filter**^42^: It is based on the concept of the Vitality Index. The Vitality Index measures the contribution of a node to the network’s modularity. It computes the modularity variation before and after removing a network node. One extracts the backbone by setting a threshold value on the node’s vitality index or selecting a top vitality fraction of nodes.
- **Planar Maximally Filtered Graph**^63^: It simply reconstructs the graph by adding edges with the highest weight iteratively as long as the resulting graph is still planar.
- **Primary Linkage Analysis**^61^: This method preserves the edge with the largest weight for each node.
- **Global Sparsification**^65^: Assuming that an edge is likely to be within the same cluster if the nodes at its endpoints have a high neighbor overlap, the algorithm calculates the similarity of the endpoints using the Jaccard similarity. Then, it extracts the backbone of the graph, applying a threshold to the edge similarity.
- **Node Degree**^64^: Node degree is computed by counting the number of connections or links a node has with other nodes in the network. One can filter the nodes based on their degree scores by setting a threshold.
- **Edge Betweenness**^62^: It computes the edge betweenness of each edge. First, it finds the shortest paths in the network. Then, for each edge, it counts the shortest paths passing through it. Edges can be filtered using a threshold based on their edge betweenness scores.

### Hybrid backbone extraction methods

The hybrid backbone extraction methods offer a unique approach by combining statistical and structural methodologies. These methods first calculate edge or node scores based on the network’s topology. Subsequently, a statistical test is applied to these computed scores.

- **Globally and Locally Adaptive Backbone**^66^: It combines the Disparity and High Salience Skeleton filters. It measures the involvement of an edge by the fraction of all the shortest paths connecting a node to the rest of the network through this edge. The edge involvement is computed at the node level. Furthermore, one uses a null hypothesis to determine the statistical significance of each edge based on its involvement. The involvement is assumed to follow a uniform Gaussian or power law distribution. A parameter regulates the influence of the node’s degree on its statistical significance.

**Table 1:** A summary of the backbone extraction method characteristics implemented in netbone.

Category	Method	Network		Filter		Parameters
		Weighted	Unweighted	Type	Scope	
Statistical	Disparity	✓	✗	Edges	Local	Alpha (significance level)
	Noise Corrected	✓	✗	Edges	Local	Alpha (significance level)
	Marginal Likelihood	✓	✗	Edges	Local	Alpha (significance level)
	Enhanced Configuration Model	✓	✗	Edges	Local	Alpha (significance level)
	Locally Adaptive Network Sparsification	✓	✗	Edges	Local	Alpha (significance level)
	Multiple Linkage Analysis	✓	✗	Edges	Local	–
Structural	Global threshold	✓	✗	Edges	Global	Threshold
	Maximum Spanning Tree	✓	✗	Edges	Global	–
	Doubly Stochastic	✓	✗	Edges	Local	Threshold
	High Salience Skeleton	✓	✗	Edges	Global	Threshold
	h-Backbone	✓	✗	Edges	Global	–
	Metric Distance Backbone	✓	✗	Edges	Global	–
	Ultrametric Distance Backbone	✓	✗	Edges	Global	–
	Planar Maximally Filtered Graph	✓	✗	Edges	Global	–
	Modularity Backbone	✓	✗	Nodes	Global	Threshold
	Primary Linkage Analysis	✓	✗	Edges	Local	–
	Global Sparsification	✓	✓	Edges	Local	Threshold
	Edge Betweenness	✓	✓	Edges	Global	Threshold
	Node Degree	✓	✓	Nodes	Global	Threshold
Hybrid	Globally and Locally Adaptive Backbone	✓	✓	Edges	Local & Global	c (involvement parameter) alpha (significance level)

Including network types (weighted/unweighted), filter type and scope (edges/nodes and local/global), and method parameters. ✓ indicate the applicability and ✗ indicate the inapplicability.

## The NetBone package

**Figure 1:**
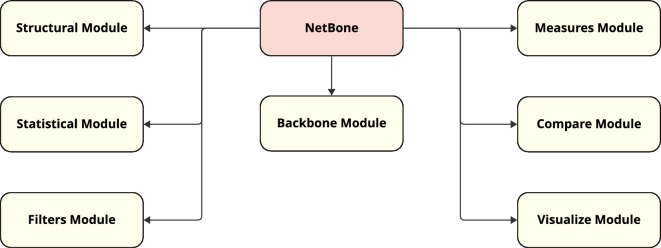
The diagram illustrates the different modules provided in the netbone package and their interactions.

Netbone is a Python package freely available to the public on GitLab (https://gitlab.liris.cnrs.fr/coregraphie/netbone). It provides a straightforward and easy-to-use framework for comparing and selecting the most appropriate method for a given case study. Figure 1 provides a diagram representing the various modules of the netbone package. We give a brief presentation of its architecture and present its various modules with their main features.

- **Backbone Module:** The Backbone module contains the Backbone class, which is central to the backbone extraction functionality. Running a backbone extraction method returns an instance of this class. It contains the calculated scores (for structural methods) or p-values (for statistical methods) associated with the nodes or edges of the chosen backbone extraction technique. One can inspect the scores or p-values by invoking the to_dataframe() function. It generates a data frame with the corresponding scores or p-values. Additionally, the module allows users to easily incorporate their newly defined backbone extraction method into the netbone comparison framework. All that is required is for the user to return an instance of the Backbone class at the end of their function.
- **Structural and Statistical Modules:** The statistical and structural modules group methods based on their underlying methodology. Invoking a function from these modules calculates p-values for statistical methods such as the disparity_filter(). It computes scores for some structural techniques such as the high_salience_skeleton(). For structural methods that extract a substructure from the graph, such as the maximum_spanning_tree(), it assigns Boolean values to the edges or nodes of the network. In all cases, it returns a new instance of the Backbone class containing the computed values.
- **Filters Module:** The filters module is a powerful component allowing users to extract the backbone that meets their specific needs. It accomplishes this by separating the backbone values calculation (score, p-value, Boolean) and the filtering process. For example, users can use the threshold_filter() function to extract a backbone based on a score or p-value threshold. They can use the fraction_filter() function to obtain a backbone of a desired size. The boolean_filter() function is designed for methods extracting a single substructure of the network. Table 2 summarizes the filters associated with the various backbone extraction methods.
- **Measures Module:**The measures module contains a set of evaluation measures allowing users to compute the topological properties of the extracted backbone. These measures have been carefully defined. They include those used in the seminal work of Serrano^45^.
- **Compare Module:** The compare module is a standout feature, providing a robust comparison framework through its Compare class. The module offers four main functions. First, the properties() function computes a set of specified properties of the original network and the extracted backbones using the desired Filter. Second, the properties_progression() function computes the evolution of given properties between the original network and a set of extracted backbones. This set can be defined using a filter type and an array of thresholds or fractions. Third, the distribution_ks_statistic() function computes the KS statistic^127^ between the cumulative distribution of an original network property and its distribution in a given backbone. It needs a function to extract the property values and a filter type. Finally, using the consent() functions, the module allows the extraction of what is called the consensual backbone. Given a set of different backbones, the method computes the intersection between the backbones
- **Visualize Module:** The visualize module is designed to facilitate the comparisons by generating visually appealing plots. It contains three main plotting functions. First, the radar_plot() function generates a radar chart with a separate axis for each added property. This chart is useful as it clearly and concisely represents multiple properties on a single plot. It simplifies the backbone’s analysis and comparison across various topological dimensions. Second, the progression_plot()function generates simple line charts that display the evolution of defined properties as the fraction or threshold changes. Finally, the distribution_plot() function generates simple scatter charts that display the distribution of defined property values in the extracted backbones.

**Table 2:** A summary of the filters that can(✓) and cannot(✗) be applied for each backbone extraction method.

Backbone extraction method	Boolean filter	Fraction filter	Threshold filter
Disparity	✗	✓	✓
Noise Corrected	✗	✓	✓
Marginal Likelihood	✗	✓	✓
Enhanced Configuration Model	✗	✓	✓
Locally Adaptive Network Sparsification	✗	✓	✓
Multiple Linkage Analysis	✓	✗	✗
Global threshold	✗	✓	✓
Maximum Spanning Tree	✓	✗	✗
Doubly Stochastic	✓	✓	✓
High Salience Skeleton	✓	✓	✓
h-Backbone	✓	✗	✗
Metric Distance Backbone	✓	✗	✗
Ultrametric Distance Backbone	✓	✗	✗
Modularity Backbone	✗	✓	✓
Planar Maximally Filtered Graph	✓	✗	✗
Primary Linkage Analysis	✓	✗	✗
Global Sparsification	✗	✓	✓
Edge Betweenness	✗	✓	✓
Node Degree	✗	✓	✓
Globally and Locally Adaptive Backbone	✗	✓	✓

## NetBone in action: a toy example

To illustrate the usage of netbone, we consider the high salience skeleton method with the Les Misérables network^128^. We chose this extraction technique because it can be associated with the three filtering methods provided by netbone. To begin using the netbone package, one can install the latest release from either the PyPI repository (https://pypi.org/project/netbone/) or directly from the project’s GitLab repository:

>
: pip install netbone
>: pip install git+https://gitlab.liris.cnrs.fr/coregraphie/netbone

Once installed, the netbone package can be imported using:

>
: import netbone as nb

The netbone package can handle two types of inputs: a networkx graph or a DataFrame. In this example, we will load the Les Misérables network from networkx and apply the high_salience_skeleton() method. The resulting scores can be examined using the to_dataframe() function as shown below:

>: import networkx as nx
>: g = nx.les_miserables_graph()
>: b = nb.high_salience_skeleton(g)
>: b.to_data_frame()

**Table:** 

Source	Target	Weight	High Salience Skeleton	Score
Napoleon	Myriel	1	True	1.000
Myriel	MlleBaptistine	8	True	0.987
Myriel	MmeMagloire	10	True	0.987
Myriel	CountessDeLo	1	True	1.000
Myriel	Geborand	1	True	1.000
Myriel	Champtercier	1	True	1.000
...	...	...	...	...

The high salience skeleton method proposed by Grady exhibits a bimodal distribution of scores centered around 0 and 1. The default approach of this method is to keep only edges with scores greater than 0.8. In netbone, it can be accomplished using the boolean_filter(). However, in that case, two nodes are missing from the extracted backbone in this particular example. To fix this issue, users can adjust the threshold by using the threshold_filter() function. One can use a threshold of 0.7 to retain all the network nodes. Additionally, users can control the size of the backbone using the fraction_filter(), such as keeping 15% of the network. The following code shows how to do it in netbone:

>
: from netbone.filters import boolean_filter, threshold_filter, fraction_filter
>: backbone1 = boolean_filter(b)
>: backbone2 = threshold_filter(b, 0.7)
>: backbone3 = fraction_filter(b, 0.15)

Once backbones are extracted, users can use them in their applications and case studies. For the sake of simplicity, we visualize the backbones using networkx in a spiral layout.

**Figure 2:**
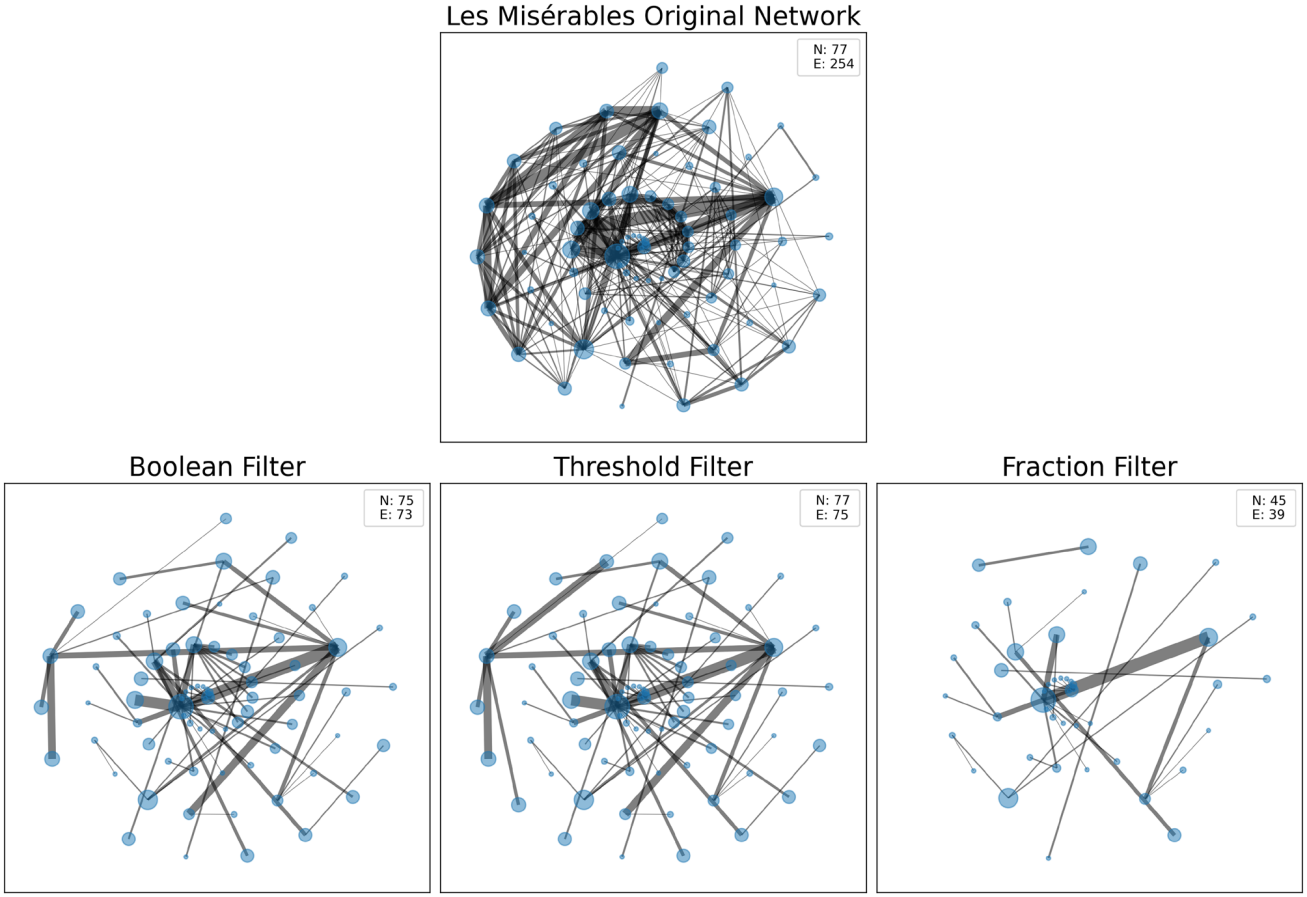
The original Les Misérables network and its extracted backbones using the boolean_filter() with a default threshold of 0.8, threshold_filter() with a threshold value of 0.7, and fraction_filter() with a fraction of 0.15. N and E are the number of nodes and edges, respectively. The size of the nodes is proportional to the degree. The width of the links is proportional to the weights.

Figure 2 presents the Les Misérables original network and its backbones using the boolean_filter(), threshold_filter(), and fraction_filter(). The size of the nodes is proportional to their degree, and the width of the link is proportional to their weights. The lower-left panel of the figure displays the backbone processed with the boolean_filter(). It is the output of the high-salience skeleton method with default values. It retains the edges that participate in at least 80% of the shortest paths in the network. The backbone is sparser than the original network, with the fraction of links reduced by 70%. However, some nodes are missing in this backbone. The lower-middle panel shows the backbone using the threshold_filter() to adjust the threshold. It includes edges participating in at least 70% of the shortest paths in the network. This backbone contains all the nodes and two more links than the previous one. The lower-right panel shows the backbone and fraction_filter() retaining only the top 15% scores of edges. It is the sparser backbone with multiple components. These edges connect 45 nodes and account for approximately 60% of all nodes in the network.

## Exploring NetBone’s comparison framework

The comparison framework of netbone stands out as a key feature. It allows users to easily explore and compare the backbones extracted from various methods with built-in evaluation measures. Moreover, users can easily integrate their backbone extraction methods and evaluation measures into the comparison framework. The framework provides five distinct use cases for comparison purposes:

The first use case involves comparing the backbone’s topological properties. It is achieved using the properties() method to compute the selected properties of the backbones. One can visualize the results in a radar plot using the radar_plot() method.

The second use case focuses on comparing the evolution of backbone properties. Users can compute the selected properties for various fractions of edges/nodes or varying significance levels or thresholds for the backbones. It can be done using the properties_progression() method. One can visualize the results in line charts using the progression_plot() method.

The third use case centers around comparing topological properties distributions. Users can assess the distances between the cumulative distributions for a given property and a couple of backbones. To do so, one must compute the Kolmogorov–Smirnov (KS) statistic of the cumulative distributions under evaluation using the distribution_ks_statistic() method. The distribution_plot() method allows visualizing the differences in a scatter plot.

The fourth use case involves extracting the consensual backbone. It entails keeping identical nodes and edges among the backbones. It can be accomplished using the consent() method.

Finally, the fifth use case illustrates how users can integrate their backbone extraction methods and their custom evaluation measures into netbone’s comparison framework.

In the following subsections, we illustrate the ability of this framework to evaluate the effectiveness of backbone extraction methods across various applications. We use the US air transportation network introduced in the work of Serrano^45^. It consists of 382 airport nodes in the continental US. The edges represent routes between these airports, and the weights assigned to the edges correspond to the number of passengers. The supplementary materials contain detailed explanations of the code for each experiment.

### Experiment 1

**Figure 3:**
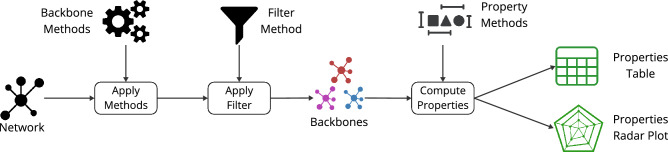
A diagram illustrating the flow within netbone’s comparison framework to compute the topological properties of the extracted backbone.

In this experiment, we focus on assessing the connectivity of the structural backbone extraction methods in the air transportation network using netbone’s comparison framework. The aim is to have a connected filtered network when applying filters since connectivity is an essential property in transportation networks. Figure 3 illustrates the process flow within netbone’s comparison framework for computing topological properties.

First, we extract from the network backbones using eight structural backbone extraction methods. We use the Boolean Filter within the framework since these methods extract a substructure from the network. Next, we compute various properties, with a particular focus on reachability. Reachability measures the connectivity between nodes in a network by quantifying the fraction of node pairs that can communicate with each other. Furthermore, we examine additional properties such as node, edge, weight fractions, density, and average degree of the extracted backbones. The results are presented in a table for easy numerical analysis and can be visualized using a spider plot.

**Figure 4:**
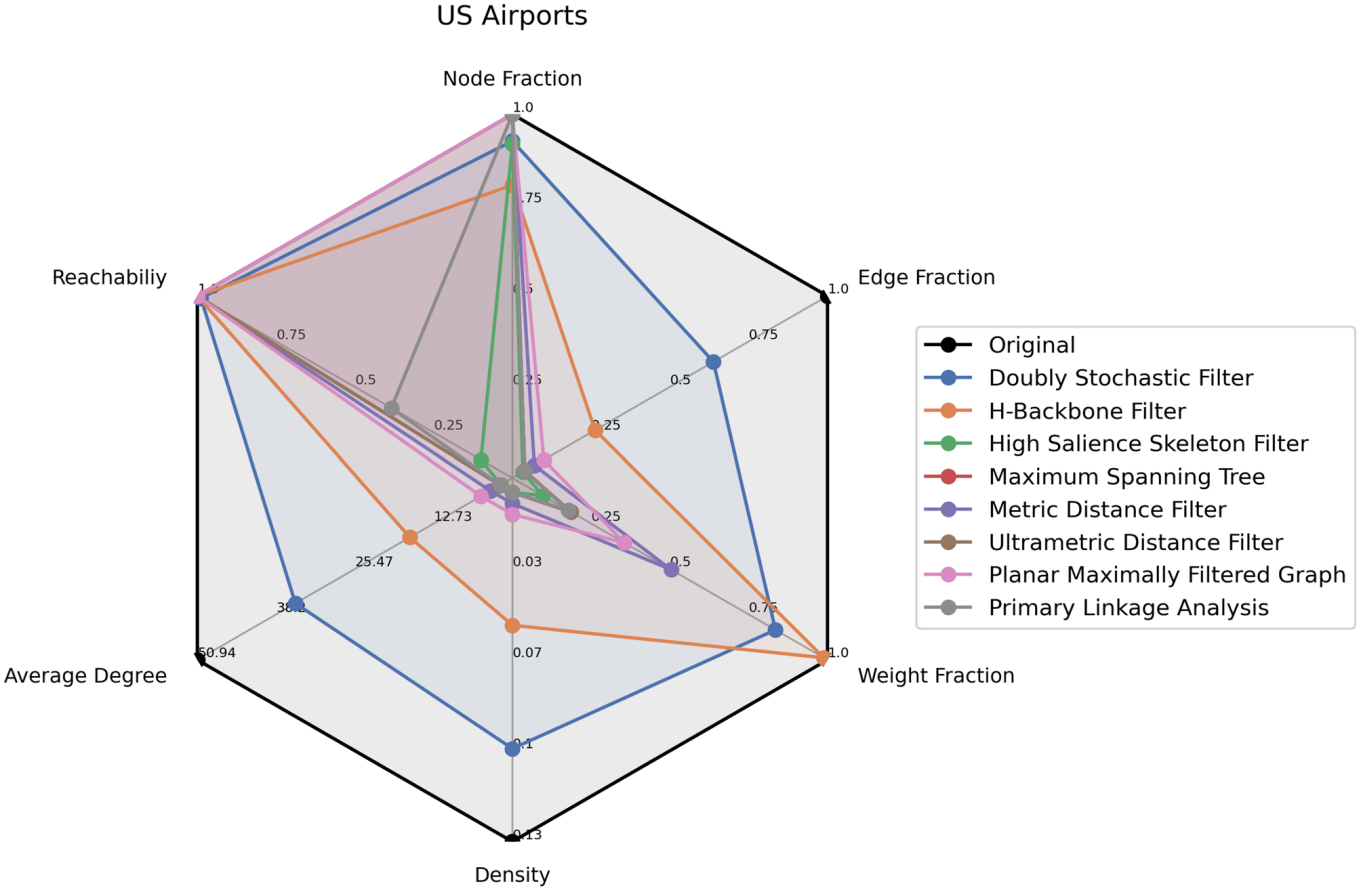
A radar chart showing the topological properties of the extracted structural backbones plotted using netbone. The topological properties are the fraction of nodes, edges, and weights preserved in the backbone, density, average degree, and reachability of the extracted backbone.

In Table 3 and Fig. 4, we present the topological properties of the extracted backbones. One can observe that all methods yield a backbone with a reachability value of 1, except for the Doubly Stochastic, Primary Link Analysis, and High Salience Skeleton methods. Reachability represents the fraction of node pairs that can communicate with each other in the network. Suppose that the user is interested in backbones with a reachability of 1. According to this criterion, one can exclude the Doubly Stochastic, Primary Link Analysis, and High Salience Skeleton methods.

**Table 3:** The topological properties of the structural backbones computed using netbone’s comparison framework.

Method	Reachability	Node fraction	Edge fraction	Weight fraction	Density	Average degree
Original Network	1	1	1	1	0.1344	50.9
h-Backbone	1	0.80	0.26	0.98	0.0544	16.5
Maximum Spanning Tree	1	1	0.03	0.18	0.0053	1.99
Metric Backbone	1	1	0.06	0.50	0.0094	3.55
Ultrametric Backbone	1	1	0.03	0.18	0.0053	1.99
Planar Maximally Graph	1	1	0.09	0.35	0.0134	5.0
Doubly Stochastic	0.98	0.92	0.63	0.83	0.1	35.0
Primary Linkage Analysis	0.38	1	0.03	0.17	0.0052	1.9
High Salience Skeleton	0.1	0.91	0.03	0.09	0.0053	1.8

Examining the Node fraction, we find that only the h-Backbone method isolates some nodes, as it preserves only 80% of the nodes. Consequently, we exclude the h-Backbone from our selection.

Moving forward, among the remaining methods, our focus shifts to choosing the technique that preserves the highest weight fraction. Consequently, we select the Metric Backbone method, which retains 50% of the weights. However, one can note that this method only includes 3% of the edges, resulting in an average degree of 3.5 and a low density of 0.0094.

To summarize, this use case filters the original transportation network under constraints. Indeed, we aim to retain all nodes while ensuring they remain connected within a single component. Furthermore, one wants to maximize the preservation of weights. Using netbone’s comparison framework, we can evaluate and compare the performance of various backbone extraction methods based on these multiple properties. It allows us to identify the Metric Backbone method as it preserved the highest fraction of weights while maintaining connectivity within a single component.

### Experiment 2

**Figure 5:**
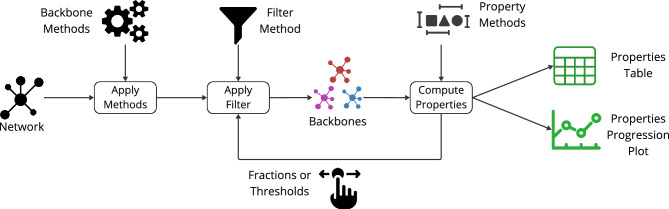
A diagram illustrating the flow within netbone comparison framework to compute the evolution of the topological properties as the threshold varies in the extracted backbone.

The Previous experiment focuses on the structural methods for backbone extraction. Some of these methods can be adjusted using a threshold on scores or selecting the top fraction of scores. In this experiment, our objective is to sparsify the network while preserving all the nodes, which is crucial in the context of a transportation network. To achieve this, we use netbone’s comparison framework to help us determine the appropriate fraction. Figure 5 illustrates the process flow within netbone’s comparison framework for computing topological properties as the fraction of edges or thresholds varies.

In the experiment, we extract the backbones using five structural backbone extraction. Using the fraction filter, we gradually sparsify the network by adjusting the fraction from 0.01 to 0.5. We aim to keep the backbone edge size below 50% of the original network. For each fraction, we compute the node fraction to assess the preservation of nodes. The results are in a table for easy analysis. Additionally, one can use a progression line plot to visualize the evolution of the node fraction as the fraction filter varies.

**Figure 6:**
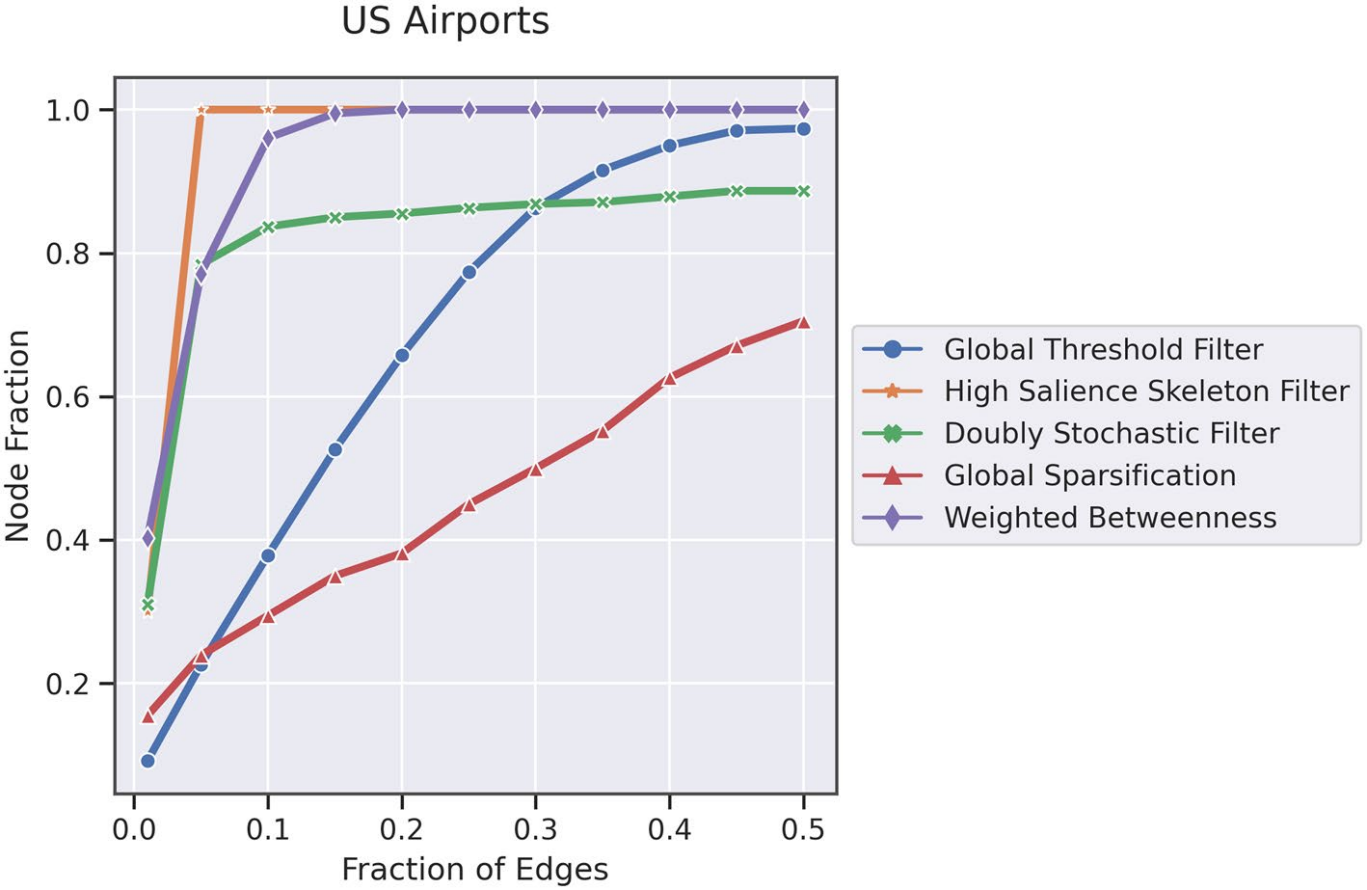
A line chart showing the fraction of nodes of the extracted structural backbones as a function of the fraction of edges plotted using netbone.

Table 4 and Fig. 6 present the node fraction for each backbone extraction method as a function of the edge fraction. We observe that the global threshold, doubly stochastic, and global sparsification methods fail to extract a backbone that includes all the nodes while keeping the edge fraction below 50%. Consequently, we can exclude these methods from our list of interests. However, the betweenness method allows us to maintain all the nodes with an edge fraction of 20%. The high salience skeleton method stands out by enabling us to preserve all the nodes with an edge fraction as low as 5%.

**Table 4:** The node fraction for each backbone extraction method as a function of the edge fraction calculated using netbone’s comparison framework.

Edge fraction	Global threshold	High salience	Doubly stochastic	Global sparsification	Weighted betweenness
0.01	0.09	0.3	0.31	0.15	0.40
0.05	0.22	**1.0**	0.78	0.23	0.77
0.10	0.37	1.0	0.83	0.29	0.96
0.15	0.52	1.0	0.85	0.35	0.99
0.20	0.65	1.0	0.85	0.38	**1.00**
0.25	0.77	1.0	0.86	0.45	1.00
0.30	0.86	1.0	0.86	0.50	1.00
0.35	0.91	1.0	0.87	0.55	1.00
0.40	0.95	1.0	0.87	0.62	1.00
0.45	0.97	1.0	0.88	0.67	1.00

Significant values are in [bold].

This experiment aims to identify the optimal structural backbone extraction method to sparsify the transportation network while preserving all the nodes. Through netbone’s comparison framework, we evaluate different methods using the fraction filter. The high salience skeleton method successfully achieved the objective by retaining all nodes with a low edge fraction of 5%. It is worth noting that one can use this approach with netbone’s comparison framework to other applications with alternative criteria.

### Experiment 3

In this experiment, we use netbone’s comparison framework to assess the global threshold and statistical methods to capture the weight and degree distributions. Indeed, using the global threshold method, the weight distribution is truncated. It emphasizes the edges between the hubs in the air transportation network. These edges typically have high weights due to the significant volume of passengers involving large carriers. One can use statistical methods to capture different scales of importance and highlight the hub and spoke topology. These methods account for multiple scales and provide a more comprehensive understanding of the network’s structure.

**Figure 7:**
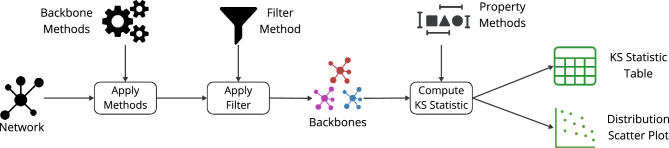
A diagram illustrating the flow within netbone’s comparison framework to compute the distribution of the topological properties in the extracted backbone.

Figure 7 illustrates the flow within the framework for comparing the cumulative distributions. First, we apply the global threshold method and statistical methods to the network. Then, we use the threshold filter within netbone’s comparison framework to filter the network. For the global threshold method, we set the threshold value to the average weight of 7000. For the statistical methods, we use a significance level of 0.05. Next, we compute the Kolmogorov–Smirnov (KS) statistic to measure the similarity between the weight and degree distributions of the original network and the backbones generated by these methods. The results are in a table for easy analysis. Additionally, one can use a distribution scatter plot to compare the distributions visually.

Table 5 presents the KS statistic for the weight and degree distributions between the original network and the extracted backbones. The Enhanced Configuration model filter exhibits the lowest KS statistic for the weight distribution. It indicates that the backbone generated by this method closely resembles the original weight distribution, effectively highlighting the hub and spoke network topology.

**Table 5:** The KS statistic comparing the weight and degree distribution between the original network and the extracted backbones was calculated using netbone’s comparison framework.

Method	Weight	Degree
Global Threshold Filter	0.80	0.40
Marginal Likelihood Filter	0.55	0.41
Noise Corrected Filter	0.51	0.54
Disparity Filter	0.70	0.49
Enhanced Configuration Model Filter	0.32	0.55
Locally Adaptive Network Sparsification Filter	0.66	0.66

**Figure 8:**
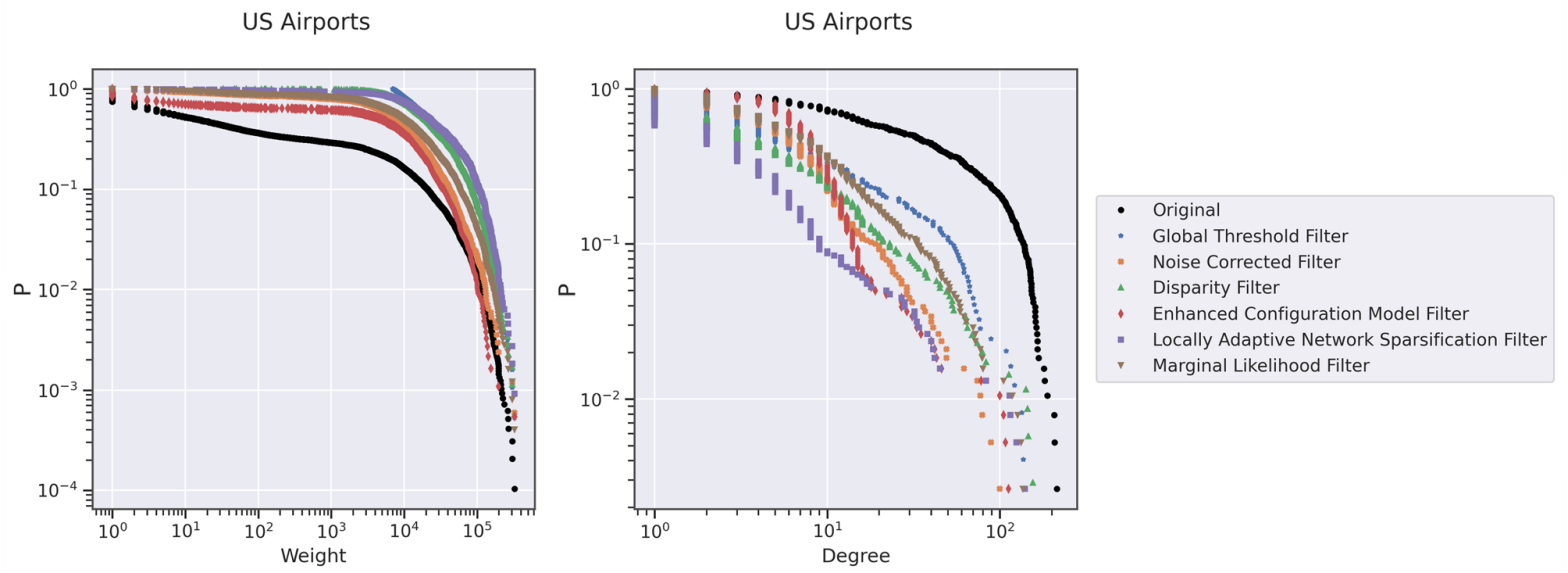
Scatter charts that display the original network’s cumulative weight and degree distribution and its extracted backbone plotted using netbone.

On the other hand, the Marginal Likelihood filter shows the lowest KS statistic for the degree distribution. This suggests that this method better preserves the degree distribution of the original network. Users seeking to retain the nearest degree distribution can consider the Marginal Likelihood filter. One can use netbone’s to plot them in a scatter plot to compare the distributions visually. Figure 8 illustrates the results of this visualization, showcasing the distributions obtained from the backbone extraction methods.

This use case involves filtering the transportation network to find a backbone with weight or degree distributions that closely match the original network. netbone’s comparison framework is crucial in selecting the most suitable backbone extraction method. Through this framework, we compare the distributions of different methods and identify the Enhance Configuration Model filter as the closest match for the weight distribution and the Marginal Likelihood filter as the closest match for the degree distribution.

### Experiment 4

The statistical methods used for backbone extraction in netbone are based on different null models, each aiming to understand the distribution or generation of weights in the network. As a result, these null models yield different backbones. netbone allows us to compute the intersection of various Backbone extraction methods. Extracting common nodes and edges across all the methods allows for observing a “consensual backbone”.

**Figure 9:**
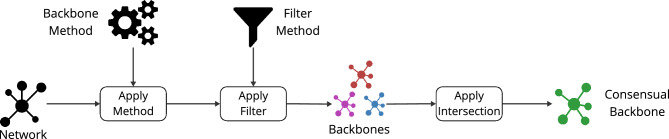
A diagram illustrating the flow within netbone’s comparison framework to extract the consensual backbone from the extracted backbones.

In this experiment, we use netbone’s comparison framework to extract the consensus backbone using the statistical backbone extraction methods. The process flow, depicted in Fig. 9, outlines the steps in extracting the consensus backbone. Firstly, we apply the statistical methods and filter them using the threshold filter with a significance level of 0.05. Then, we extract the consensus backbone by taking the intersection of the extracted backbones.

**Figure 10:**
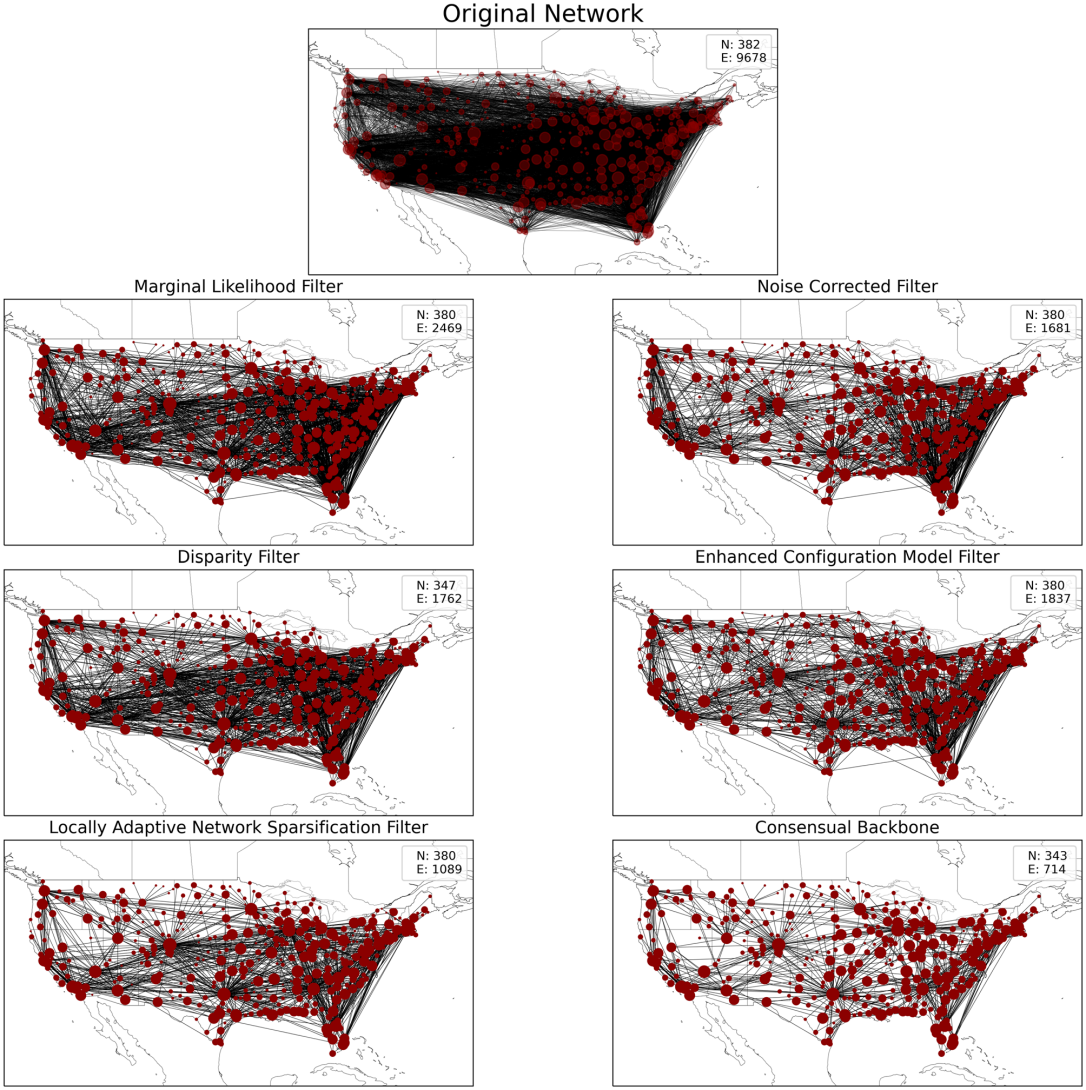
The US air transportation network and its corresponding backbones, extracted using netbone. N and E are the number of nodes and edges, respectively.

Figure 10 provides a visual representation of the extracted backbones, showcasing the number of preserved nodes and edges in each method. The consensual backbone includes 343 nodes and 714 edges. These nodes and edges hold significant value when considering the various null models used by the statistical methods. Comparing the consensual backbone to the other techniques, we can observe that it prominently highlights the hub and spoke network structure more effectively than the individual statistical methods.

This experiment demonstrates that netbone’s statistical consensual backbone effectively emphasizes the hub and spoke network structure compared to individual statistical backbones. Moreover, it’s worth noting that the consensual extraction method is not restricted to statistical methods. Users can also use it with structural methods, allowing for various combinations and variations of backbones to explore and analyze the distinctive characteristics of these consensual backbones.

### Experiment 5

This experiment illustrates how users can integrate their custom backbone extraction method and custom evaluation properties into netbone’s comparison framework. To illustrate this process, we define the new_backbone_method() function. It generates random values and keeps them in a new edge property named new_score. The function should return a new instance of the Backbone class. To initialize an instance of the Backbone class, users should provide a networkx graph containing the new edge scores, the name of the new method, the edge property name. If the edge property name represents a p-value, it should be set to True. Otherwise, it should be False. Next, users must specify an array of compatible filters. Given that the edge property is a numerical value, the appropriate filters to use in this case are the threshold_filter and the fraction_filter. Lastly, the filter_on parameter should indicate whether the filter is applied to edges or nodes.
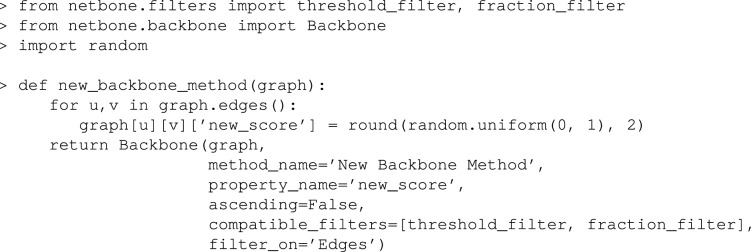


Once the new backbone extraction method is defined, one can easily apply the method and add it to the comparison framework using the add_backbone() method. Users can now continue by adding the evaluation measures from the built-in methods in netbone or by implementing their new custom evaluation measure. To illustrate this, we define the new_property_method() method. This method will imitate the node_fraction() method; it returns the node fraction preserved in the backbone. The method should take two inputs, the original and the backbone graphs. And it should return the computed property value.
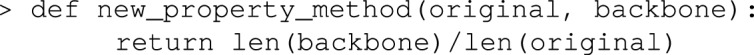


## Conclusion

In conclusion, netbone is a powerful, free, open-source Python package. It offers a variety of statistical and structural methods for extracting network backbones. Its filters can meet all use cases, and the comparison framework is a standout feature. It enables users to compare backbones with a wide range of evaluation measures. The Five experiments conducted in this paper illustrate the wide range of possible scenarios that can be analyzed using netbone. The first experiment showcases how the comparison framework can assist in evaluating the backbone extraction methods by comparing various topological properties. The second experiment highlights how the framework could aid users in determining the appropriate fraction or threshold for extracting backbones. The third experiment illustrates how users evaluate the distribution of property values of the extracted backbones. The fourth experiment introduces the consensual backbone and how users can create unlimited combinations of the backbone techniques. Finally, the fifth experiment illustrates how users can integrate their backbone extraction methods and custom evaluation measures into the comparison framework. Overall, the comparison framework provides users a valuable tool for comparing backbone extraction methods. In its current developmental phase, our primary focus has been on integrating classical backbone extraction methods for unweighted and weighted networks into the package. However, many situations are well described by bipartite networks. For example, in social networks, one can connect users with events. In recommendation systems, users are linked to items. Converting these bipartite graphs into a static network by projections removes important information. Tumminello, Neal, and others^29,36^ have proposed backbone extraction techniques specifically designed for bipartite projections to address this limitation. A key objective is to extend the netbone package by incorporating these specialized approaches. Another crucial extension concerns temporal networks. Indeed, aggregating network snapshots into a static representation entails a substantial loss of information. It can lead to conventional classical methods of weighted backbone extraction neglecting significant aspects of the underlying network. Kobayashi and others^67^ have introduced backbone extraction methods for temporal networks in response to this challenge. Consequently, our second goal is to enrich further the netbone package by incorporating these advanced techniques. Lastly, in a third major development direction, we aim to expand the scope of netbone to Multilayer networks. We believe these extensions will provide a comprehensive package enabling handling a broader network analysis range.

## Data and methods

This section introduces the data and methods used in the toy example and the three experiments to evaluate the backbone extraction methods.

### Data

In this subsection, we introduce the networks used in the experiments, Table 6 reports their basic topological features.

#### Les Misérables

In the Les Misérables Network^128^, nodes represent actors in Victor Hugo’s novel. They are connected if they appear in the same chapter of the Les Misérables novel. Edge weights denote the number of such occurrences.

#### US air transportation

In the US Air Transportation Network^45^, nodes represent airports in the continental US, and edges represent the routes between these airports. Edge weights correspond to the number of passengers for the year 2006.

**Table 6:** The Topological features of the Les Misérables and US Air Transportation networks.

Network	N	E	${<k>}$	$\rho$
Les Misérables	77	254	6.5	0.087
US Air Transportation	380	9678	50.9	0.134

N is the number of nodes. |*E*| is the number of edges. $<k>$ is the average degree. $\rho$ is the density.

### Methods

In this subsection, we present the evaluation measures used in the experiments to evaluate the extracted backbones.

#### Node fraction

The node fraction in the backbone represents the proportion of nodes retained from the original network.

#### Edge fraction

The edge fraction in the backbone represents the proportion of edges retained from the original network.

#### Weigh fraction

The weight fraction in the backbone represents the proportion of edge weights retained from the original network.

#### Average degree

The average degree is the sum of the degrees of all network nodes divided by the number of nodes in the network.

#### Density

The density is the ratio between the edges present in a network and the maximum number of edges that the network can contain.

#### Reachability

The Reachability^129^ quantifies the connectivity between any pair of nodes in a network. It is defined as the fraction of node pairs that can communicate with each other. This reads:

1 $$\begin{aligned} R = \frac{1}{n(n-1)}\sum _{i\ne j \in G}{R_{ij}}. \end{aligned}$$

with *n* is the number of nodes and $R_{ij} = 1$ if path exists between node *i* and *j* and $R_{ij}=0$ otherwise. The Reachability values are in the [0, 1] range. If any pair of nodes can communicate in a network, the reachability *R* becomes 1. If $R=0$ it means all nodes are isolated from each other.

#### Two-sample Kolmogorov–Smirnov

The two-sample Kolmogorov–Smirnov test (KS test)^127^ allows testing whether two samples follow the same distribution. Simply put, the KS statistic for the 2-sample test is the greatest distance between each sample’s CDFs (Cumulative Distribution Function). Thus, the Kolmogorov–Smirnov statistic *D* is given by:

2 $$\begin{aligned} D_{m,n} = \max _{x}|F(x)-G(x)| \end{aligned}$$

where *F*(*x*) and *G*(*x*) represent the CDF of the two samples, and *n* and *m* are the numbers of observations of the first and second samples, respectively.

### Supplementary Information

Supplementary Information.

## Data Availability

All data used in the toy example and the experiments are available at https://gitlab.liris.cnrs.fr/coregraphie/netbone/tree/main/examples/data.

## Code availability

NetBone is distributed via the pypi package index (https://pypi.org/project/netbone/) and is developed publically on GitLab (https://gitlab.liris.cnrs.fr/coregraphie/netbone). The examples section in the repository contains the code required to reproduce the results presented in this manuscript.
