# Supplementary material for: An evaluation tool for backbone extraction techniques in weighted complex networks
Source: Sci Rep. 2023 Oct 9;13:17000. doi: 10.1038/s41598-023-42076-3 (PMC10562457; doi:10.1038/s41598-023-42076-3)
Supplement: Supplementary file 1 — Supplementary Information. [file 41598_2023_42076_MOESM1_ESM.pdf]

An Evaluation Tool for Backbone Extraction Techniques in Weighted Complex Networks

July 5, 2023

1 Requirements

netbone is available on Pypi. But make sure you have Python version 3.10 or higher and it's a good idea to use conda, virtualenv, or pyenv.

!pip install netbone

Once installed, the *netbone* package can be imported simply

[1]:
```
import netbone as nb
```

2 Toy Example

To cover all users needs we separated the calculation process from the filtering process in *netbone*. Thus, the process of extracting the backbone follows: 1. Apply a backbone extraction method to run the computation process 2. Apply a filter to extract the backbone

To illustrate the usage of *netbone*, we consider the high salience skeleton method with the Les Misérables network. We chose this extraction technique because it can be associated with the three filtering methods provided by *netbone*. The *netbone* package can handle two types of inputs: a *networkx* graph or a *DataFrame*. In this example, we will load the Les Misérables network from *networkx* and apply the *high_ salience_ skeleton()* method.

[2]:
```
import networkx as nx
g = nx.les_miserables_graph()

b = nb.high_salience_skeleton(g)
```

The resulting scores can be examined using the *to_ dataframe()* function as shown below:

[3]:
```
b.to_dataframe()
```

[3]:

	source	target	weight	distance	in_backbone	salience
0	Napoleon	Myriel	1	1.000000	True	1.000000
1	Myriel	MlleBaptistine	8	0.125000	True	0.987013
2	Myriel	MmeMagloire	10	0.100000	True	0.987013
3	Myriel	CountessDeLo	1	1.000000	True	1.000000
4	Myriel	Geborand	1	1.000000	True	1.000000

```
..             ...            ...   ...       ...         ...       ...
249          Babet         Brujon     3  0.333333       False  0.025974
250     Claquesous    Montparnasse    2  0.500000       False  0.025974
251     Claquesous         Brujon     1  1.000000       False  0.000000
252   Montparnasse         Brujon     1  1.000000       False  0.000000
253         Child1         Child2     3  0.333333       False  0.025974

[254 rows x 6 columns]
```

The high salience skeleton method exhibits a bimodal distribution of scores centered around 0 and 1. The default approach of this method is to keep only edges with scores greater than 0.8. In *netbone*, it can be accomplished using the *boolean_filter()*. However, in that case, two nodes are missing from the extracted backbone in this particular example. To fix this issue, users can adjust the threshold by using the *threshold_filter()* function. One can use a threshold of 0.7 to retain all the network nodes. Additionally, users can control the size of the backbone using the *fraction_filter()*, such as keeping 15% of the network. The following code shows how to do it in *netbone*:

[4]:
```
from netbone.filters import boolean_filter, threshold_filter, fraction_filter

backbone1 = boolean_filter(b)
backbone2 = threshold_filter(b, 0.7)
backbone3 = fraction_filter(b, 0.15)
```

```
High Salience Skeleton Filter
High Salience Skeleton Filter
High Salience Skeleton Filter
```

To illustrate the usage of the extracted backbones, we plot them using *netowrkx*.

[5]:
```
import matplotlib.pyplot as plt

fig = plt.figure(figsize=(18, 12),tight_layout=True)
rows = 2
columns = 3
node_scale = 10
edge_scale = 0.5

deg = nx.degree(g)
pos = nx.spiral_layout(g)

grid = plt.GridSpec(rows, columns, wspace = .025, hspace = .1)
sizes = [node_scale * deg[n] for n in g.nodes()]
weg = [edge_scale * g[u][v]['weight'] for u,v in g.edges()]

ax = plt.subplot(grid[0,1:2])

ax.set_title('Les Misérables Original Network', fontsize=20)
nx.draw_networkx_nodes(g,  pos=pos, nodelist=['Child1'], node_color='white',␣
↪node_size=[node_scale * deg[n] for n in ['Child1']], alpha=0.01)
```

```
nx.draw_networkx(g, ax=ax,
                 alpha=.5,
                 # width=.6,
                 node_size=sizes,
                 width = weg,
                 # node_color='k',
                 pos=pos,
                 with_labels=False,
                 font_size=50)
plt.legend([f'E: {len(g.edges())} \nN: {len(g.nodes())}'], handlelength=0, handleheight=0)

titles = ['Boolean Filter', 'Threshold Filter', 'Fraction Filter']
for i, backbone in enumerate([backbone1, backbone2, backbone3]):
    sizes = [node_scale * deg[n] for n in backbone.nodes()]
    weg = [edge_scale * backbone[u][v]['weight'] for u,v in backbone.edges()]


    ax = plt.subplot(grid[1,i])

    ax.set_title(titles[i], fontsize=20)
    removed = g.nodes() - backbone.nodes()
    nx.draw_networkx_nodes(g,  pos=pos, nodelist=removed, node_color='white', node_size=[node_scale * deg[n] for n in removed], alpha=0.0)
    nx.draw_networkx_nodes(g,  pos=pos, nodelist=['Child1'], node_color='white', node_size=[node_scale * deg[n] for n in ['Child1']], alpha=0.0)
    nx.draw_networkx(backbone, ax=ax,
                     alpha=.5,
                     # width=.6,
                     node_size=sizes,
                     width = weg,
                     # node_color='k',
                     pos=pos,
                     with_labels=False)
    # plt.legend([r'$\bf{N}$' + f': {len(backbone.nodes())} \n' + r'$\bf{E}$' + f': {len(backbone.edges())}'], handlelength=0, handleheight=0)
    plt.legend([f'E: {len(backbone.edges())} \nN: {len(backbone.nodes())}'], handlelength=0, handleheight=0)

# plt.savefig('./images/toy.pdf', dpi=300, bbox_inches='tight')
plt.savefig('./images/toy.png', dpi=300, bbox_inches='tight', transparent=True)
#
```

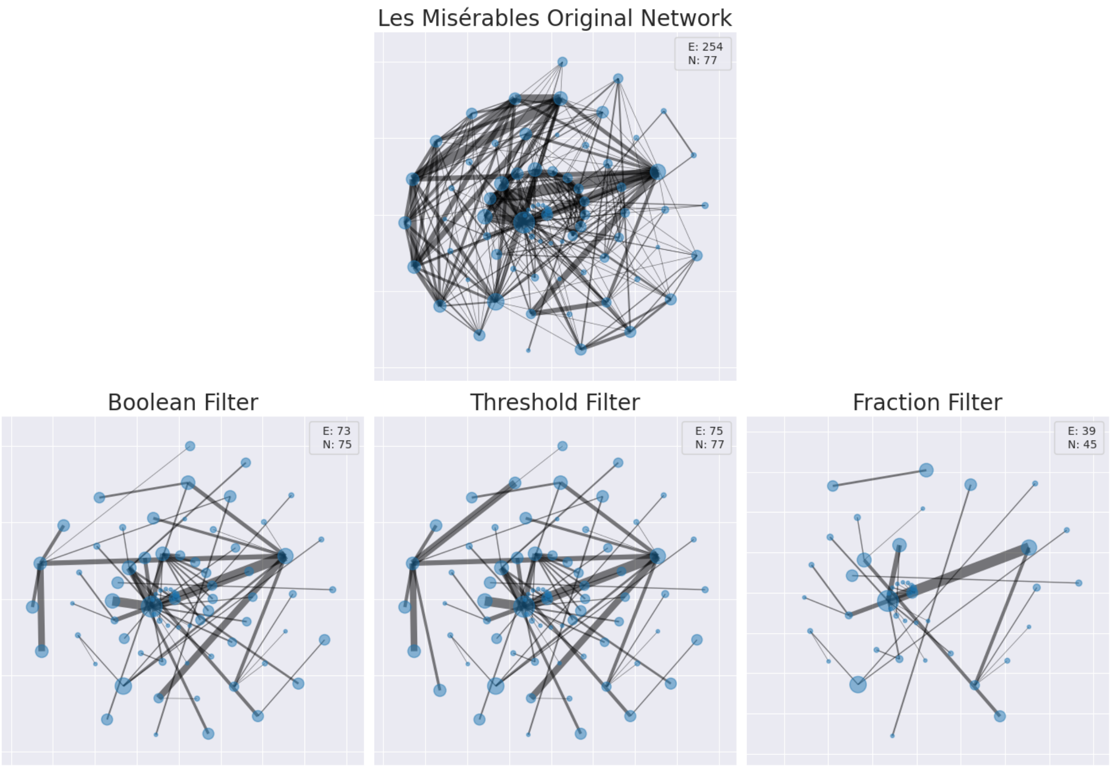

3 Experiment 1

In this experiment, we focus on assessing the connectivity of the structural backbone extraction methods in the air transportation network using *netbone*'s comparison framework. The aim is to have a connected filtered network when applying filters since connectivity is an essential property in transportation networks. To accomplish this, first we define an instance of the *Compare* class from the *compare* module.

[6]:
```
from netbone.compare import Compare
framework = Compare()
```

After initialization, the first step is to add the original network to *netbone*'s comparison framework using the *set_network()* function. For this purpose, we must provide a *networkx* graph or an edge list stored in a *DataFrame* object. In this experiment, we use a *DataFrame* object.

[7]:
```
import pandas as pd
edge_list = pd.read_csv('./data/data.csv')
framework.set_network(edge_list)
```

The next step is to set up the filter in the comparison framework. It is done using the *set_filter()* function. It specifies the filter used to extract the backbones before computing the properties. In this experiment, we choose to use the *boolean_filter()*. Since the selected methods extract one subgraph by there definition.

[8]:
```
from netbone.filters import boolean_filter
framework.set_filter(boolean_filter)
```

After setting the original network and filter, the next step is to add the backbone extraction methods to the comparison framework. This is done in two stages, first we apply the backbone extraction method. Then we add them to the comparison framework using the add_backbone() function. Here we chose to use eight structural techniques. We recall that in *netbone* the computation process is separated of the filtration process. Subsequently, the backbone extraction method in *netbone* returns an instance of the *Backbone* Class.

[9]:
```
import netbone as nb
ds = nb.doubly_stochastic(edge_list)
hb = nb.h_backbone(edge_list)
hss = nb.high_salience_skeleton(edge_list)
msp = nb.maximum_spanning_tree(edge_list)
mb = nb.metric_distance_backbone(edge_list)
umb = nb.ultrametric_distance_backbone(edge_list)
pmfg = nb.pmfg(edge_list)
pla = nb.plam(edge_list)


framework.add_backbone(ds)
framework.add_backbone(hb)
framework.add_backbone(hss)
framework.add_backbone(msp)
framework.add_backbone(mb)
framework.add_backbone(umb)
framework.add_backbone(pmfg)
framework.add_backbone(pla)
```

The final step is to add the properties used to evaluate the backbones. To add a property, users can use the *add_property()* function by passing it a name and a property function. Here, we use six predefined property functions from the *measures* module

[10]:
```
from netbone.measures import node_fraction, edge_fraction, average_degree,␣
 ↪reachability, weight_fraction, density
framework.add_property('Node Fraction', node_fraction)
framework.add_property('Edge Fraction', edge_fraction)
framework.add_property('Weight Fraction', weight_fraction)
framework.add_property('Density', density)
framework.add_property('Average Degree', average_degree)
framework.add_property('Reachability', reachability)
```

Now that everything is set up and added to the framework, we call the *properties()* function to compute the added properties. This function returns a pandas *DataFrame* that can be inspected to compare the computed properties of the backbones

[11]:
```
results = framework.properties()
results
```

[11]:

	Node Fraction	Edge Fraction
Original	1.000000	1.000000
Doubly Stochastic Filter	0.926316	0.638045
H-Backbone Filter	0.805263	0.262244
High Salience Skeleton Filter	0.918421	0.033478
Maximum Spanning Tree	1.000000	0.039161
Metric Distance Filter	1.000000	0.069746
Ultrametric Distance Filter	1.000000	0.039161
Planar Maximally Filtered Graph	1.000000	0.099711
Primary Linkage Analysis	1.000000	0.038748

	Weight Fraction	Density	Average Degree
Original	1.000000	0.1344	50.936842
Doubly Stochastic Filter	0.834884	0.1000	35.085227
H-Backbone Filter	0.988625	0.0544	16.588235
High Salience Skeleton Filter	0.096433	0.0053	1.856734
Maximum Spanning Tree	0.186046	0.0053	1.994737
Metric Distance Filter	0.503583	0.0094	3.552632
Ultrametric Distance Filter	0.186046	0.0053	1.994737
Planar Maximally Filtered Graph	0.355704	0.0134	5.078947
Primary Linkage Analysis	0.177826	0.0052	1.973684

	Reachability
Original	1.000000
Doubly Stochastic Filter	0.988669
H-Backbone Filter	1.000000
High Salience Skeleton Filter	0.100007
Maximum Spanning Tree	1.000000
Metric Distance Filter	1.000000
Ultrametric Distance Filter	1.000000
Planar Maximally Filtered Graph	1.000000
Primary Linkage Analysis	0.384294

To perform the comparative analysis of backbone extraction techniques visually, we plot the properties across various dimensions using a *radar_plot()* function from the *visualize* module. This function takes two inputs: the results *DataFrame* and a *String* representing the title of the figure and the name of the saved figure file.

[12]:
```
from netbone.visualize import plot_radar
plot_radar(results, 'US Airports')
```

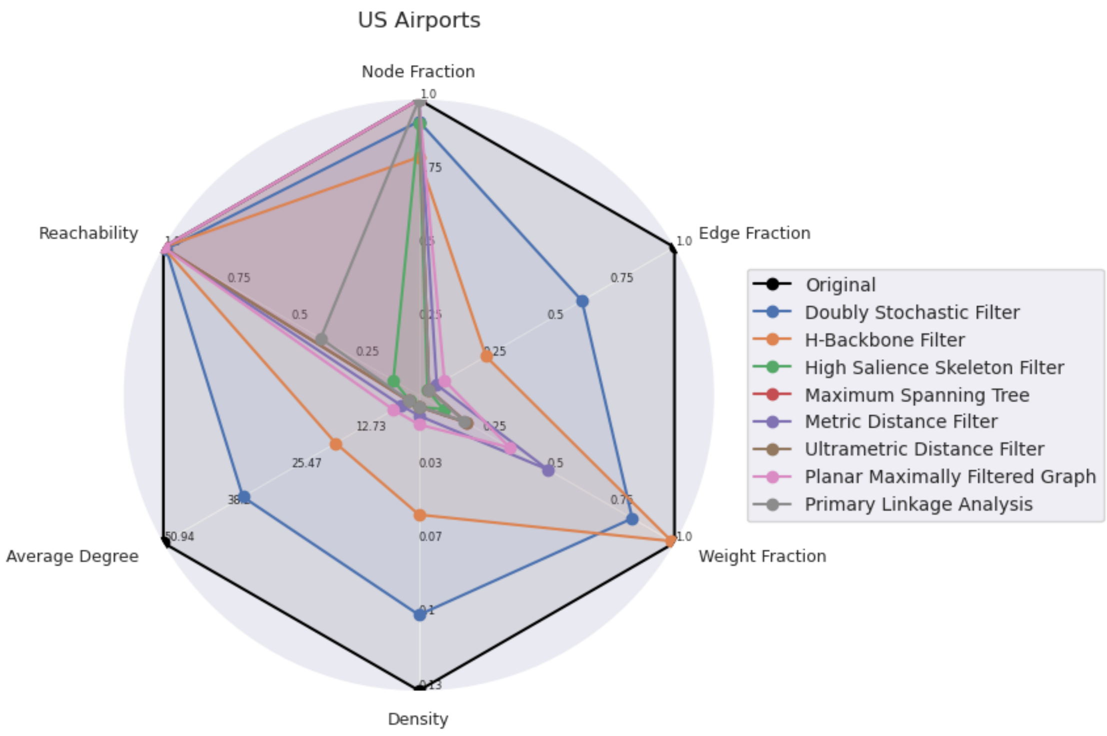

4 Experiment 2

The Previous experiment focuses on the structural methods for backbone extraction. Some of these methods can be adjusted using a threshold on scores or selecting the top fraction of scores. In this experiment, our objective is to sparsify the network while preserving all the nodes, which is crucial in the context of a transportation network. To achieve this, we use *netbone*'s comparison framework to help us determine the appropriate fraction. We start by initiating an instance of the Compare class from the compare module. Then we add the original network to *netbone*'s comparison framework using the *set_network()* function.

[13]:
```
from netbone.compare import Compare
import pandas as pd

framework = Compare()

edge_list = pd.read_csv('./data/data.csv')
framework.set_network(edge_list)
```

The next step is to set up the filter in the comparison framework. In this experiment, we choose to use the *fraction_filter()* to evaluate the backbones at the fractions from 0.01 till 0.5. Thus, we pass an array of these values while setting the filter.

[14]:
```
from netbone.filters import fraction_filter

fractions = [0.01, 0.05, 0.1, 0.15, 0.2, 0.25, 0.3, 0.35, 0.4, 0.45, 0.5]
framework.set_filter(fraction_filter, fractions)
```

Once the original network and filter are set, the following step is to add the backbone extraction methods in the comparison framework.

[15]:
```
import netbone as nb

gt =nb.global_threshold(edge_list)
hss = nb.high_salience_skeleton(edge_list)
ds = nb.doubly_stochastic(edge_list)
gspar = nb.gspar(edge_list)
bet = nb.betweenness(edge_list, weighted=True)

framework.add_backbone(gt)
framework.add_backbone(hss)
framework.add_backbone(ds)
framework.add_backbone(gspar)
framework.add_backbone(bet)
```

The last step is incorporating the properties to assess the backbones under varying fractions. In this case, we use one property function, the *node_fraction()* from the *measures* module.

[16]:
```
from netbone.measures import node_fraction

framework.add_property('Node Fraction', node_fraction)
```

After configuring everything and adding it to the framework, the next step is to call the *properties_progression()* function to compute the properties for the backbone at each fraction. The output of this function is a *dictionary* of *DataFrame*s. One can use it to inspect the computed properties of the backbones with respect to the fractions.

[17]:
```
results= framework.properties_progression()
results['Node Fraction']
```

[17]:
```
                    Global Threshold Filter  High Salience Skeleton Filter  \
Fraction of Edges
0.01                               0.092105                            0.3
0.05                               0.226316                            1.0
0.10                               0.378947                            1.0
0.15                               0.526316                            1.0
0.20                               0.657895                            1.0
0.25                               0.773684                            1.0
0.30                               0.863158                            1.0
0.35                               0.915789                            1.0
0.40                               0.950000                            1.0
0.45                               0.971053                            1.0
```

```
0.50                                0.973684                            1.0

                   Doubly Stochastic Filter  Global Sparsification  \
Fraction of Edges
0.01                                0.310526               0.155263
0.05                                0.784211               0.239474
0.10                                0.836842               0.294737
0.15                                0.850000               0.350000
0.20                                0.855263               0.381579
0.25                                0.863158               0.450000
0.30                                0.868421               0.500000
0.35                                0.871053               0.552632
0.40                                0.878947               0.626316
0.45                                0.886842               0.671053
0.50                                0.886842               0.705263

                   Weighted Betweenness
Fraction of Edges
0.01                           0.402632
0.05                           0.771053
0.10                           0.960526
0.15                           0.994737
0.20                           1.000000
0.25                           1.000000
0.30                           1.000000
0.35                           1.000000
0.40                           1.000000
0.45                           1.000000
0.50                           1.000000
```

To visualize the evolution of the properties versus the fraction values, we use the *plot_progression()* function from the *visualize* module. This function requires two arguments: the results *dictionary* and a *String* that represents the title of the figure and the name of the saved figure file.

[18]:
```
from netbone.visualize import plot_progression
plot_progression(results, 'US Airports')
```

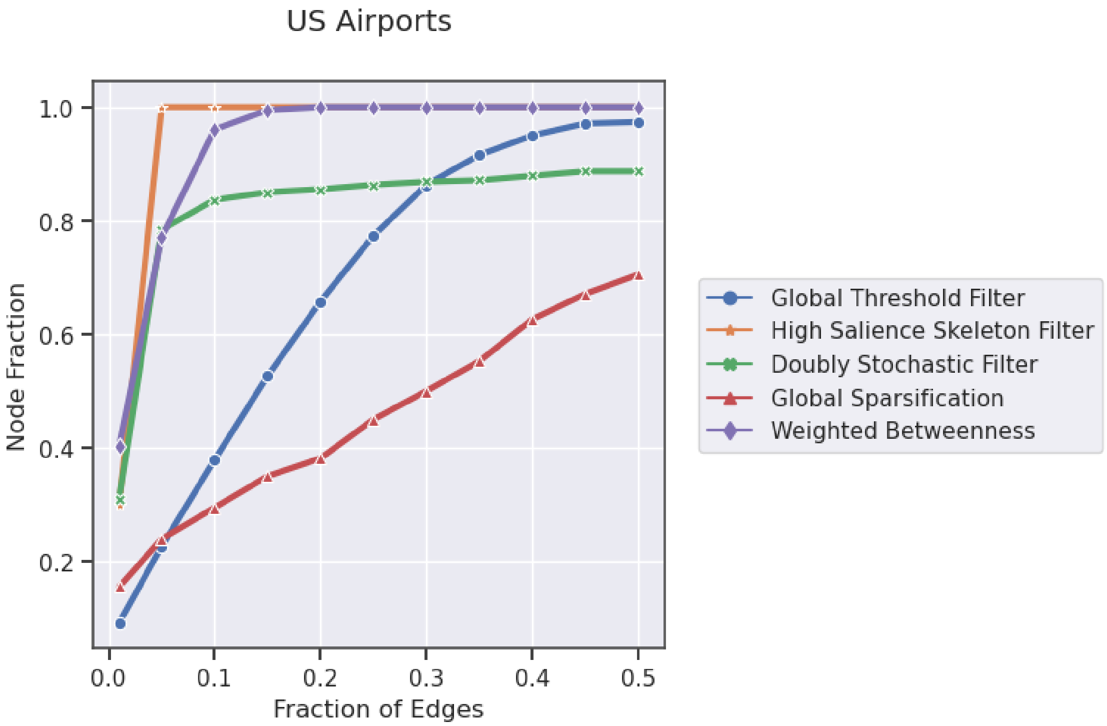

5 Experiment 3

In this experiment, we use *netbone*'s comparison framework to assess the global threshold and statistical methods to capture the weight and degree distributions. We start by initiating an instance of the Compare class from the compare module. Then we add the original network to *netbone*'s comparison framework using the *set_network()* function.

[19]:
```
from netbone.compare import Compare
import pandas as pd

framework = Compare()

edge_list = pd.read_csv('./data/data.csv')
framework.set_network(edge_list)
```

Then we add the backbone extraction methods to the comparison framework. Here, the order is important because we are going to use the order of the added backbones in the next step.

[20]:
```
import netbone as nb
gt = nb.global_threshold(edge_list)
df = nb.disparity(edge_list)
mlf = nb.marginal_likelihood(edge_list)
nc = nb.noise_corrected(edge_list)
```

```
ecm = nb.ecm(edge_list)
lans = nb.lans(edge_list)

framework.add_backbone(gt)
framework.add_backbone(nc)
framework.add_backbone(df)
framework.add_backbone(ecm)
framework.add_backbone(lans)
framework.add_backbone(mlf)
```

The next step is to set up the filter in the comparison framework. In this experiment, we choose to use the *threshold_filter()* to evaluate the backbones. For the global threshold method, we set the threshold value to the average weight of 7000. For the statistical methods, we use a significance level of 0.05. Thus, we pass an array of these values while setting the filter taking into consideration the order when we added the backbones.

[21]:
```
from netbone.filters import threshold_filter

values = [7000] + [0.05]*5
framework.set_filter(threshold_filter, values)
```

The last step is incorporating the property functions that will extract the values to assess the distribution of the properties in the backbones. In this case, we use two property functions, the *weights()* and *degrees()* from the *measures* module.

[22]:
```
from netbone.measures import weights, degrees

framework.add_property('Weight', weights)
framework.add_property('Degree', degrees)
```

After configuring everything and adding it to the framework, the next step is to call the *distribution_ks_statistic()* function to compute the KS statistic between the original and backbone property distributions. The output of this function is a *DataFrame* and a *dictionary.* One can use the *DataFrame* to inspect the computed KS statistic for each property, and the *dictionary* is used later for visualization.

[23]:
```
results, dist = framework.distribution_ks_statistic()
results
```

[23]:

	Weight	Degree
Global Threshold Filter	0.805125	0.406337
Noise Corrected Filter	0.517305	0.542105
Disparity Filter	0.700747	0.494889
Enhanced Configuration Model Filter	0.325893	0.555263
Locally Adaptive Network Sparsification Filter	0.662704	0.665789
Marginal Likelihood Filter	0.553501	0.415789

To visualize the cumulative distribution of the properties, we use the *plot_distribution()* function from the visualize module. This function requires two arguments: the results dictionary and a

String that represents the title of the figure and the name of the saved figure file.

[24]:
```
from netbone.visualize import plot_distribution
plot_distribution(dist, title='US Airports')
```

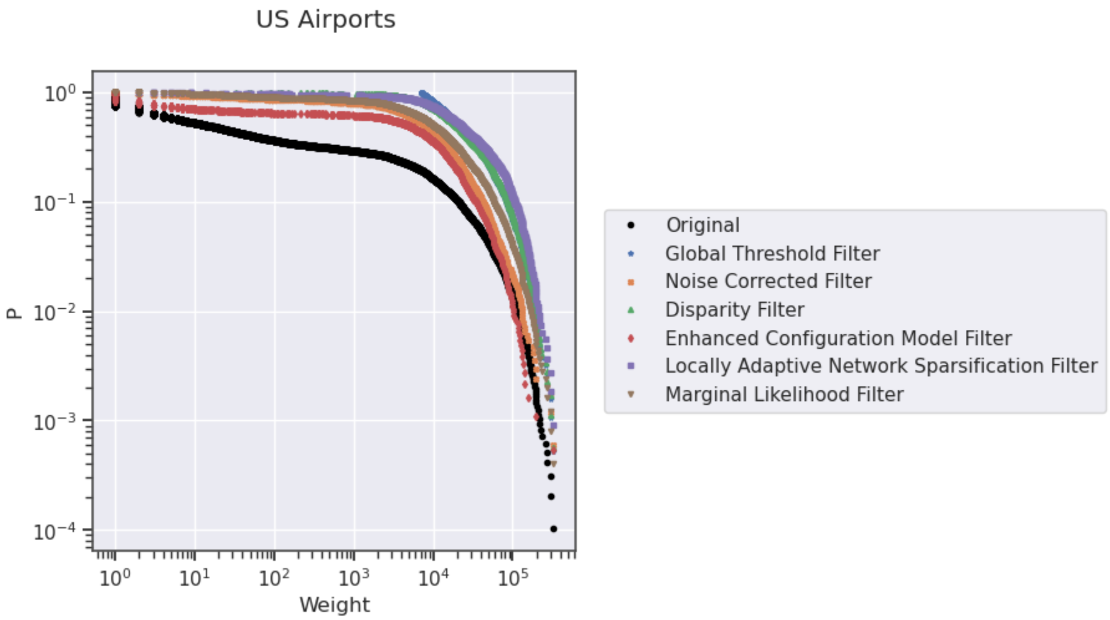

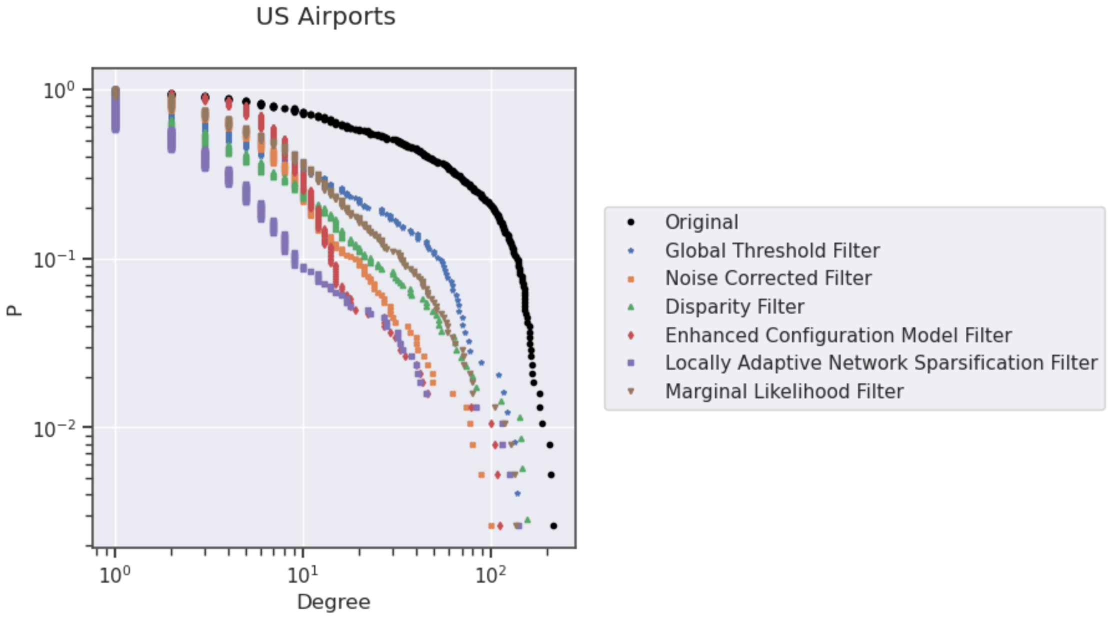

6 Experiment 4

In this experiment, we use *netbone*'s comparison framework to extract the consensus backbone using the statistical backbone extraction methods. We start by initiating an instance of the Compare class from the compare module. Then we add the original network to *netbone*'s comparison framework using the *set_network()* function.

[25]:
```
from netbone.compare import Compare
import pandas as pd

framework = Compare()

edge_list = pd.read_csv('./data/data.csv')
framework.set_network(edge_list)
```

Then we add the backbone extraction methods to the comparison framework. Similar to the previous experiment, the order is important because we are going to use the order of the added backbones in the next step.

[26]:
```
import netbone as nb

df = nb.disparity(edge_list)
mlf = nb.marginal_likelihood(edge_list)
nc = nb.noise_corrected(edge_list)
ecm = nb.ecm(edge_list)
lans = nb.lans(edge_list)

framework.add_backbone(nc)
framework.add_backbone(df)
framework.add_backbone(ecm)
framework.add_backbone(lans)
framework.add_backbone(mlf)
```

The next step is to set up the filter in the comparison framework. In this experiment, we choose to use the *threshold_filter()* to evaluate the backbones. We set the threshold value to 0.05. Thus, we pass an array of these values while setting the filter taking into consideration the order when we added the backbones.

[27]:
```
from netbone.filters import threshold_filter

values = [0.05]*5
framework.set_filter(threshold_filter, values)
```

Here we don't need to add any property function we simply use the method *consent()*. By taking the intersection of the extracted backbones, this method returns a *netowrkx* graph representing the consensus backbone.

[28]:
```
consensual = framework.consent()
```

Next we extract the backbones similar to the toy example to prepare it for plotting later.

[29]:
```
mlf_backbone = threshold_filter(mlf, 0.05)
nc_backbone = threshold_filter(nc, 0.05)
df_backbone = threshold_filter(df, 0.05)
ecm_backbone = threshold_filter(ecm, 0.05)
lans_backbone = threshold_filter(lans, 0.05)

backbones = [mlf_backbone, nc_backbone, df_backbone, ecm_backbone,␣
↪lans_backbone, consensual]
b = [mlf.method_name, nc.method_name, df.method_name, ecm.method_name, lans.
↪method_name, 'Consensual Backbone']
```

```
Marginal Likelihood Filter
Noise Corrected Filter
Disparity Filter
Enhanced Configuration Model Filter
Locally Adaptive Network Sparsification Filter
```

We extract the coordinates of the nodes and the degree of each node to plot the nodes in the right position.

[30]:
```
import pyreadr
import networkx as nx
result = pyreadr.read_r('./data/data.RData')
g = nx.from_pandas_adjacency(result['airport'])
positions = {index: tuple(row) for index, row in result['latlong'].iterrows()}
deg = nx.degree(g)
```

We plot the original network with the backbones using cartopy and networkx.

[31]:
```
import cartopy.crs as ccrs
import cartopy.feature as cfeature
import matplotlib.pyplot as plt
import seaborn as sns
sns.reset_defaults()
crs = ccrs.PlateCarree()
fig = plt.figure(figsize=(18, 20))
rows = 5
columns = 2


grid = plt.GridSpec(rows, columns, wspace = .025, hspace = .1)

sizes = [.5 * deg[iata] for iata in g.nodes()]
ax = plt.subplot(grid[0,:], projection=crs)
ax.coastlines(lw=0.2)

ax.set_extent([-128, -62, 20, 50])
ax.add_feature(cfeature.BORDERS, color="k", lw=0.2)
```

```
ax.add_feature(cfeature.STATES, lw=0.1)
ax.set_title('Original Network', fontsize=20)
ax.set_aspect('equal')
nx.draw_networkx(g, ax=ax,
                 alpha=.5,
                 width=.3,
                 node_size=sizes,
                 node_color='#8b0000',
                 pos=positions,
                 cmap=plt.cm.autumn,
                 with_labels=False,
                 edge_color='k')
ax.legend([f'N: {len(g.nodes())} \nE: {len(g.edges())}'], handlelength=0,␣
 ↪handleheight=0, markerscale=0)

# b = ['Marginal Likelihood', 'Noise Corrected', 'Disparity Filter', "ECM␣
 ↪Filter", "LANS Filter", 'Consensual Backbone', 'Global Threshold']
for i, ax in enumerate(backbones):
    ax = plt.subplot(grid[int(i/2)+1,i%2], projection=crs)


    backbone = backbones[i]
    sizes = [.5 * deg[iata] for iata in backbone.nodes()]

    ax.coastlines(lw=0.2)
    ax.set_extent([-128, -62, 20, 50])
    ax.add_feature(cfeature.BORDERS, color="k", lw=0.2)
    ax.add_feature(cfeature.STATES, lw=0.1)

    ax.set_title(b[i], fontsize=14)
    ax.set_aspect('equal')
    nx.draw_networkx(backbone, ax=ax,
                     # alpha=.5,
                     width=.3,
                     node_size=sizes,
                     node_color='#8b0000',
                     pos=positions,
                     cmap=plt.cm.autumn,
                     with_labels=False,
                     edge_color='k')
    # nx.draw_networkx_nodes(backbone, pos=positions, nodelist=backbone.
 ↪nodes()['ALB'], node_color='white', alpha=0.0)
    ax.legend([f'N: {len(backbone.nodes())} \nE: {len(backbone.edges())}'],␣
 ↪handlelength=0, handleheight=0, markerscale=0)

plt.show()
plt.savefig('networks+consenual.png', dpi=300, bbox_inches='tight')
```

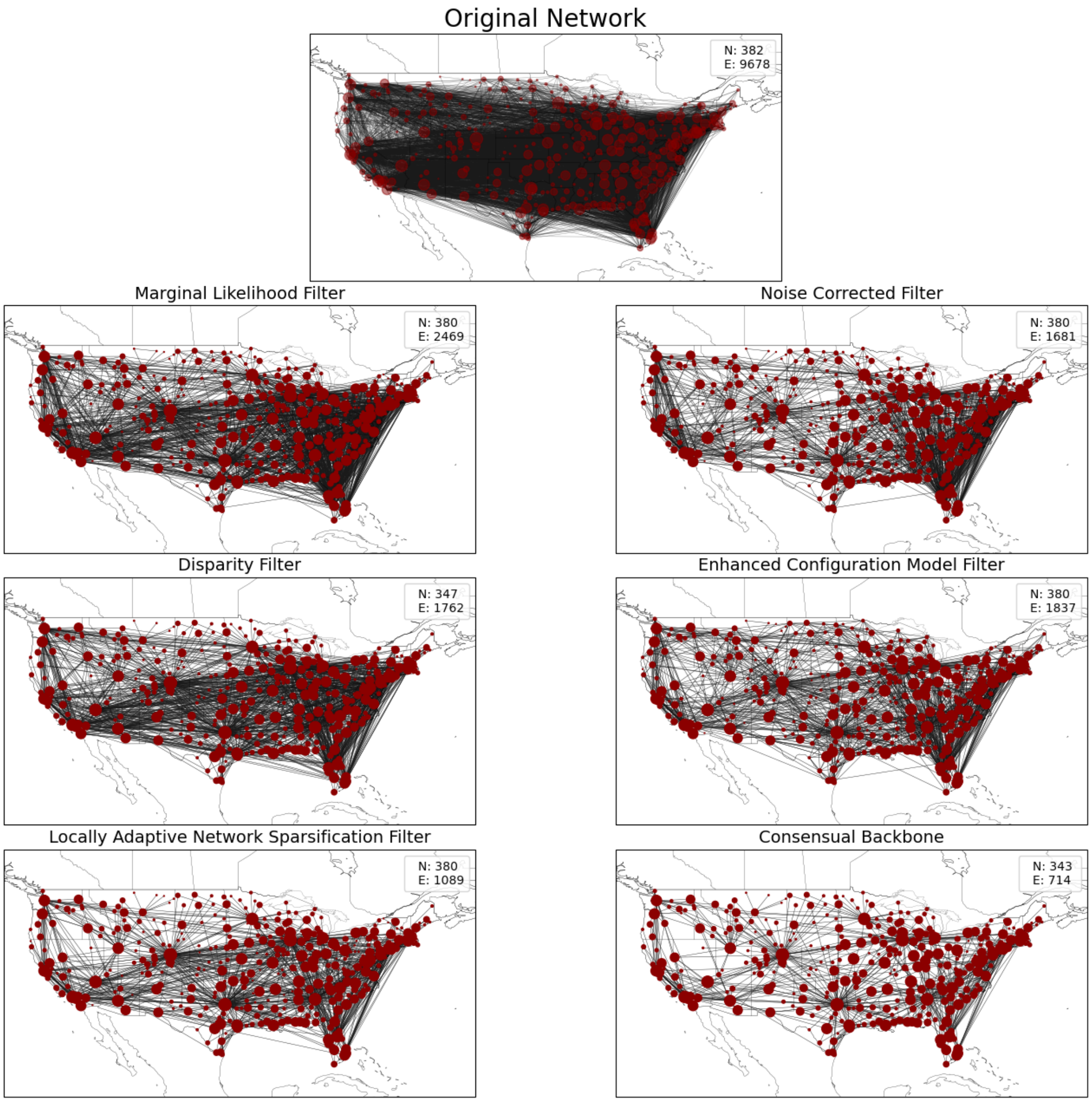

7 Experiment 5

This experiment illustrates how users can integrate their custom backbone extraction method and custom evaluation properties into *netbone*'s comparison framework. To illustrate this process, we define the *new_ backbone_ method()* function. It generates random values and keeps them in a new edge property named *new_ score*. The function should return a new instance of the *Backbone* class. To initialize an instance of the *Backbone* class, users should provide: 1. *networkx* graph containing the new edge scores 2. The name of the new method 3. The edge property name 4. The ascending parameter: It should be set to *True* if the edge property name represents a p-value. Otherwise, it should be *False* 5. An array of compatible filters. Here, the edge property is a numerical value then the appropriate filters to use in this case are the *threshold_ filter()* and the *fraction_ filter()* 6. The *filter_ on* parameter: should indicate whether the filter is applied to 'Edges' or 'Nodes'.

[32]:
```
from netbone.filters import threshold_filter, fraction_filter
from netbone.backbone import Backbone
import random

def new_backbone_method(graph):
    for u,v in graph.edges():
        graph[u][v]['new_score'] = round(random.uniform(0, 1), 2)
    return Backbone(graph, method_name='New Backbone Method',␣
↪property_name='new_score', ascending=False,␣
↪compatible_filters=[threshold_filter, fraction_filter], filter_on='Edges')
```

netbone allows users to implement their new custom evaluation measure. To illustrate this, we define the *new_property_method()* method. This method will imitate the *node_fraction()* method; it returns the node fraction preserved in the backbone. The method should: 1. Take two inputs: (the original and backbone networks) 2. Return the computed property value.

[33]:
```
def new_property(original, backbone):
    return len(backbone)/len(original)
```

Once the new backbone extraction method and evaluation measures are defined. One can easily add integrate them into the comparison framework using the *add_backbone()* and *add_property()* methods. The following example illustrates comparing the new defined method with the Disparity filter in terms of the new defined property.

[34]:
```
from netbone.filters import threshold_filter
from netbone.compare import Compare
from netbone.utils.utils import edge_properties
import pandas as pd
import netbone as nb

framework = Compare()

edge_list = pd.read_csv('./data/data.csv')
graph = nx.from_pandas_edgelist(edge_list, edge_attr=edge_properties(edge_list))
framework.set_network(edge_list)

thresholds = [0.05, 0.9]
framework.set_filter(threshold_filter, thresholds)

df = nb.disparity(graph)
new = new_backbone_method(graph)

framework.add_backbone(df)
framework.add_backbone(new)

framework.add_property('New Property', new_property)
framework.properties()
```

[34]:

	New Property
Original	1.000000
Disparity Filter	0.913158
New Backbone Method	0.792105

Users also can compare different distributions. To illustrate this, we define a new method named *distribution_property()*. It will imitate the *weights()* method; it returns all the edge weights in the backbone. The method should: 1. Take one inputs: the backbone network 2. Return and array of the computed property values

[35]:
```
def distribution_property(backbone):
    return list(nx.get_edge_attributes(backbone, 'weight').values())
```

One can easily add integrate them into the comparison framework using the add_property() method. The following example illustrates comparing the new defined method with the Disparity filter in terms of the new defined distribution property.

[36]:
```
from netbone.filters import threshold_filter
from netbone.compare import Compare
import pandas as pd
import netbone as nb
from netbone.utils.utils import edge_properties
import networkx as nx
framework = Compare()

edge_list = pd.read_csv('./data/data.csv')
graph = nx.from_pandas_edgelist(edge_list, edge_attr=edge_properties(edge_list))

framework.set_network(edge_list)

thresholds = [0.05, 0.98]
framework.set_filter(threshold_filter, thresholds)

df = nb.disparity(graph)
new = new_backbone_method(graph)

framework.add_backbone(df)
framework.add_backbone(new)

framework.add_property('Distribution Property', distribution_property)

results, dist = framework.distribution_ks_statistic()
results
```

[36]:

	Distribution Property
Disparity Filter	0.700747
New Backbone Method	0.078684
